# Supplementary material for: Peer-led counselling with problem discussion therapy for adolescents living with HIV in Zimbabwe: A cluster-randomised trial
Source: PLoS Med. 2022 Jan 5;19(1):e1003887. doi: 10.1371/journal.pmed.1003887 (PMC8730396; doi:10.1371/journal.pmed.1003887)
Supplement: S1 Text — (PDF) [file pmed.1003887.s002.pdf]

CIFF-funded cluster-randomised trial of community-based Friendship Bench to improve adherence and mental health among children and adolescents living with HIV in Zimbabwe

Analysis Plan

1. Introduction

Whilst 80% of the estimated 64,000 adolescents living with HIV (ALHIV) in Zimbabwe have been initiated on ART, a recent study of 500 adolescents found that 48% had virological failure. Globally, adolescents are the age group with the highest rate of HIV treatment failure, and the only age group in which HIV-related mortality is not slowing. ALHIV face many challenges to adherence including mental health disorders.

The Zvandiri programme, developed by Africaid, is a model of differentiated clinical service delivery for children, adolescents and young people in Zimbabwe. At the forefront of Zvandiri are trained, mentored peer supporters living with HIV known as Community Adolescent Treatment Supporters (CATS) who are 18-24 years old. CATS are integrated within the clinics and surrounding communities and generate demand for HIV services. This CATS model results in improved adherence, retention and virological suppression among ALHIV compared with adolescents receiving standard of care alone.

The Friendship Bench is a low-intensity mental health intervention delivered by trained and supervised lay health workers. It is based on cognitive behavioral therapy principles with an emphasis on problem-solving therapy (PST). Zvandiri aims to adopt Friendship Bench for its CATS by training and mentoring them in problem-solving therapy.

The overall goal of the study is to generate evidence around the feasibility and effectiveness of a unique, peer-led mental health support intervention, which seeks to improve both virological suppression and mental health in ALHIV (age 10-19 years old). The aim is to evaluate whether training and mentoring the existing CATS in problem-solving therapy (CATS-PST) will, compared with standard CATS care, reduce virological failure and common mental disorders among ALHIV in Zimbabwe.

2. Hypotheses

- The primary hypothesis is that the CATS-PST intervention will be more effective than standard CATS care in reducing the proportion of ALHIV who have died or who have virological failure at 48 weeks (defined as a viral load ≥1000 copies/ml).
- The secondary hypotheses are that adolescents receiving the CATS-PST intervention will have (i) reduced prevalence and severity of depression and/or anxiety (using the SSQ-14 and PHQ-9 scales) and (ii) improved health-related quality of life, compared with adolescents receiving standard CATS care at 48 weeks.

Objectives

The trial will provide evidence on whether training and mentoring the existing CATS in problem-solving therapy will, compared with standard CATS care:

i) reduce the proportion of adolescents with virological failure or death at 48 weeks (primary outcome);
ii) reduce prevalence and severity of depression and/or anxiety at 48 weeks;

iii) improve quality of life

3. Trial design

The trial is being conducted in ten districts in five provinces. The trial is a cluster randomized controlled trial. The clinic is the unit of randomization. Sixty clinics were randomized to either enhanced ART adherence support through the Zvandiri programme or CATS-PST in a 1:1 allocation stratified by district. Adolescents aged 11-19 years were recruited. Trial outcomes are assessed through a clinical, behavioural and psychological assessment conducted at baseline and after 48 weeks. The primary endpoint will be at 48 weeks post-enrolment, with an 8-week window period either side (ie 40-56 weeks).

4. Eligibility criteria

i) Inclusion criteria: HIV positive adolescents, aged 11-19 years, eligible for ART (i.e. either starting or already on ART) and able to provide informed assent and their caregiver is able to provide informed consent (those aged 18 and 19 years do not need caregiver consent).

ii) Exclusion criteria: Too physically or psychologically unwell to participate; or unable to give informed consent.

Outcomes

The **primary outcome** is the proportion who have died or have a viral load $\geq$1000 copies/mL at 48 weeks, assessed on a dried blood spot sample.

Secondary outcomes are assessed at 48 weeks, and are the proportion of participants

i. with common mental disorders (depression and/or anxiety)
ii. with poor quality of life

Sample size and potential power of the trial

The total sample size of 840 participants recruited from 60 clusters will provide 85% power to detect a difference in virological non-suppression of 36% among participants in the standard care arm versus 24% in the intervention arm assuming 20% loss to follow-up and a coefficient of variation (k) between clusters of 0.25. For secondary outcomes, the sample size provides 87% power to detect a difference in the proportion with CMD at 12 months of 16% in the standard CATS arm and 8% in the CATS-PST arm.

5. Quantitative data analysis

The data will be analysed using Stata 15 or above. Statistical analyses will be conducted and reported in line with Consolidated Standards of Reporting Trials (CONSORT) guidelines (extended for cluster randomised trials[1] including a flowchart. Analysis programs ("do-files") will be prepared based on blinded data, and unblinding will not take place until after analysis and interpretation of blinded results has taken place.

5.1. Recruitment and representativeness of recruited patients and patients who completed follow up

Initial analyses will compare baseline characteristics of i) individuals who consented and did not consent, and ii) participants who did and did not complete outcome assessments at 48 weeks, compared using mixed-effects logistic regression (linear regression for continuous variables) with a random effect for clustering. CONSORT flow charts will be constructed (see appendix 1).

5.2. Baseline comparability of randomised groups

Baseline characteristics of enrolled participants will be compared between treatment arms and also reported overall, summarised using mean and standard deviation, median and interquartile range or numbers and proportions as appropriate. No significance testing will be done as any differences are due to chance if randomisation was correctly applied. For continuous outcomes, histograms will also be plotted within each arm to assess normality, and whether any transformation is required.

Among those enrolled, the following baseline characteristics will be compared by arm:

- age
- gender
- in school
- orphanhood status
- HIV viral load at enrolment
- aware of HIV status
- ever disclosed HIV status
- mean SSQ score
- mean PHQ-9 score

Any variables for which there is a substantial imbalance will be noted so that final analysis can take this into account.

5.3. Adherence to allocated intervention and intervention fidelity

To assess treatment fidelity in the CATS arm, the quantity/coverage of the active treatment will be described, as indicated by number of support group meetings attended as well as home visits and SMS reminders received.

5.4. Loss to follow-up and other missing data

The numbers and proportions actively withdrawing from the trial and passively lost to follow-up will be reported overall and by arm at 48 weeks (window period 40-56 weeks). The reasons for withdrawal from the trial will be summarised.

5.5. Description of counsellors

The CATS will be described in terms of age, gender, education and caseload.

6. Outcome analysis

6.1. Description of cohort at follow up

Selected characteristics of those seen and not seen at 48 weeks will be compared to assess generalisability of results. Attrition will be compared by arm to assess bias due to loss to follow-up, using mixed-effects logistic regression to adjust for within-clinic clustering. Variables associated with missing endline data will be adjusted for in subsequent analyses. Reasons for not attending follow up will be analysed by arm. The outcome measures will be summarised at baseline and 48 weeks by intervention arm, using proportions or means (SD) as appropriate.

6.2. Analysis of intervention effect

The primary analyses will be intention-to-treat, adjusted for baseline values of the outcome measure and for variables that are imbalanced between arms at baseline. The primary analysis will be complete case, with appropriate methods to impute missing outcome data as sensitivity analyses if necessary. Analyses will be based on individual-level measures, as there are sufficient clusters per arm to allow them to perform robustly, even with stratification by district [2].

For binary outcomes, a logistic regression random effects model will be used to estimate the odds ratio of the outcome, with a random effects term to allow for clustering by clinic. A quadrature check will be performed to evaluate the model fit. For continuous outcomes a mixed effects linear regression model will be used to estimate the mean difference between arms with a random effect to allow for clustering.

Pre-defined sensitivity analyses will include adjustment for baseline viral load and other variables imbalanced at baseline. We will conduct sensitivity analyses to investigate the effect of missing data by using multiple imputation, analysed using an individual level Poisson regression model, and a per-protocol analysis.

6.3. Analysis of primary outcome

The **primary outcome** is the proportion who have died or have a viral load $\geq$1000 copies/mL at 48 weeks (virological nonsuppression), assessed on a dried blood spot sample. This will be analysed using odds ratios as described above.

6.4. Analysis of secondary outcomes

Secondary outcomes are assessed at 48 weeks, and are the proportion of participants:

Outcome	Definition	Notes	Reference
Depression	Score of ≥8/27 on the Patient Health Questionnaire (PHQ-9). Severity will be assessed using PHQ-9 as an ordinal score.	Scale has been widely used in Africa, and recently validated for use in Zimbabwe among young people living with HIV (Rabia Khan, unpublished)	[3,4,5]
Common mental disorders	Score of ≥8/14 on the Shona Symptom Questionnaire (SSQ-14);<br>Severity of CMD will be assessed using SSQ-14 as a continuous score		[6]
Poor quality of life	Measured using a previously-validated Shona version of the EQ-5D scale, analysed as a continuous score		[7,8]

7. Statistical considerations

Adjustment for multiple outcomes and reporting p-values

No p-value adjustment will be conducted. Interpretation of the intervention effect will be based on the strength of evidence of effect size and consistency of results for related outcomes.

Missing baseline and outcome data

The number (%) of participants with complete data will be reported. If scales have recommended methods for dealing with missing data, these will be applied. As outlined above, primary analyses will be complete case, with adjustments made for baseline variables which are associated with the outcome and/or missingness, to account for missing data. If necessary, in sensitivity analyses, we will apply appropriate methods to impute missing outcome data (see below).

Model assumption checks

For continuous outcomes, model residuals will also be plotted to check for normality and inspected for outliers. If substantial departures from normality occur, transformations will be considered. If a suitable transformation cannot be found, a non-parametric analysis will be considered.

Programme records

Programme data will be collected in intervention communities and regularly compiled to record uptake and attendance at community intervention, visits by CATS and use of clinical services.

References

1. Campbell, M.K., et al., *Consort 2010 statement: extension to cluster randomised trials.* BMJ, 2012. 345: p. e5661.
2. Hayes, R. and L. Moulton, *Cluster Randomised Trials*. 2009, Boca Raton: Taylor & Francis.

3. Kroenke, K., R.L. Spitzer, and J.B. Williams, *The PHQ-9: validity of a brief depression severity measure.* J Gen Intern Med, 2001. 16(9): p. 606-13.
4. Monahan, P.O., et al., *Validity/reliability of PHQ-9 and PHQ-2 depression scales among adults living with HIV/AIDS in western Kenya.* J Gen Intern Med, 2009. 24(2): p. 189-97.
5. Chibanda, D., et al., *Validation of screening tools for depression and anxiety disorders in a primary care population with high HIV prevalence in Zimbabwe.* J Affect Disord, 2016. 198: p. 50-5.
6. Patel, V., et al., *The Shona Symptom Questionnaire: the development of an indigenous measure of common mental disorders in Harare.* Acta Psychiatr Scand, 1997. 95(6): p. 469-75.
7. Jelsma, J., et al., *The reliability of the Shona version of the EQ-5D.* Cent Afr J Med, 2001. 47(1): p. 8-13.
8. Jelsma, J., et al., *The Validity of the Shona Version of the EQ-5D Quality of Life Measure.* South African Journal of Physiotherapy, 2002. 58(3): p. 8-12.

Figure 1

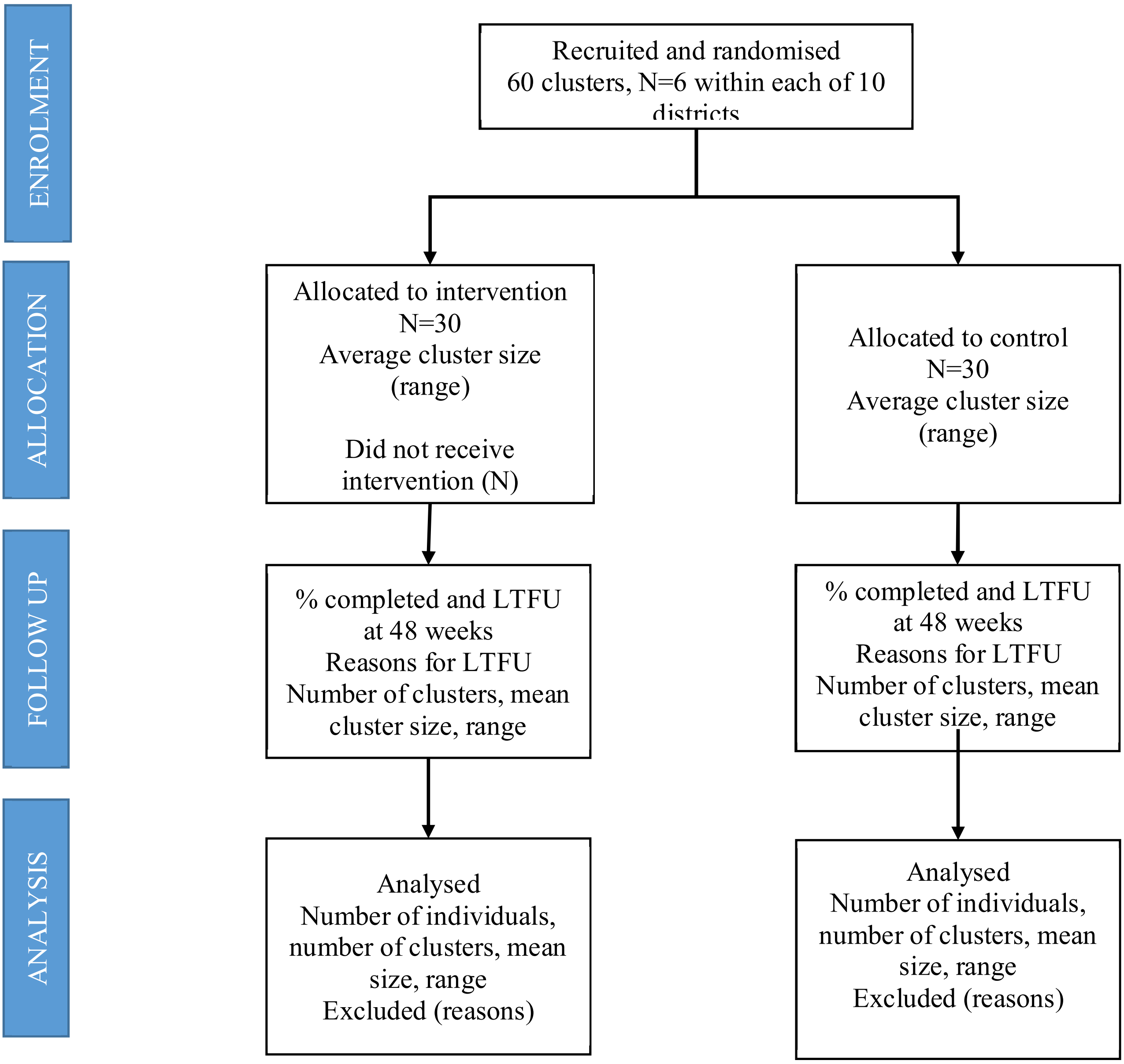
ENROLMENT
ALLOCATION
FOLLOW UP
ANALYSIS
Recruited and randomised
60 clusters, N=6 within each of 10 districts
Allocated to intervention
N=30
Average cluster size (range)
Did not receive intervention (N)
Allocated to control
N=30
Average cluster size (range)
% completed and LTFU at 48 weeks
Reasons for LTFU
Number of clusters, mean cluster size, range
% completed and LTFU at 48 weeks
Reasons for LTFU
Number of clusters, mean cluster size, range
Analysed
Number of individuals, number of clusters, mean size, range
Excluded (reasons)
Analysed
Number of individuals, number of clusters, mean size, range
Excluded (reasons)

Table 1: Baseline descriptive characteristics

		**Arm X**	**Arm Y**
		N (%)	N (%)
Gender	Male<br>Female		
Age group	13-14<br>15-16<br>17-19		
Currently in school	Yes<br>No		
Marital status	Married<br>Living with partner<br>Not living with partner<br>Single		
Orphanhood status	Not orphan<br>Maternal<br>Paternal<br>Both		
Number of children in household	<=1<br>2<br>3<br>>=4		
Disclosed HIV status	No<br>Yes<br>Don't know		
Mean SSQ score (sd)			
SSQ score	<8<br>>=8		
Mean PHQ-9 score (sd)			
PHQ-9 score	<10<br>>=10		
HIV viral load	<1000 c/ml<br>≥1000 c/ml		

Table 2: Impact of intervention on virological failure, psychological distress and stigma

	Baseline		Endline				
Outcome	Arm X	Arm Y	Arm X	Arm Y	Intervention effect		p-value
	% with outcome[1]	% with outcome[1]	% with outcome[1]	% with outcome[1]	Adjusted OR (95% CI)		
**Primary outcome**							
VF or death							
Depression (PHQ9 score >=8)							
Common mental disorders (SSQ-14 score >=8)							
	Mean (SE)	Mean (SE)	Mean (SE)	Mean (SE)	Adjusted mean difference (95% CI)	Standardised mean difference (95%CI)	
Depression (PHQ9 score)							
Common mental disorders (SSQ-14 score)							
Poor quality of life (EQ-5D)							

1 Geometric mean of the cluster-level proportions
