# Supplementary material for: Peer-led counselling with problem discussion therapy for adolescents living with HIV in Zimbabwe: A cluster-randomised trial
Source: PLoS Med. 2022 Jan 5;19(1):e1003887. doi: 10.1371/journal.pmed.1003887 (PMC8730396; doi:10.1371/journal.pmed.1003887)
Supplement: S1 Table — (DOCX) [file pmed.1003887.s003.docx]

### S1 Table: Association of baseline characteristics with missing primary outcome

|  |  | **No viral load at endline, n/N (%)** | **Crude** | | **Age-adjusted** | |
| --- | --- | --- | --- | --- | --- | --- |
|  |  |  | **OR (95% CI)** | **P** | **OR (95% CI)** | **p** |
|  | *N* | *88/842 (10.5)* |  |  |  |  |
| **Gender** | Male | 36/375 (9.6) | 1 |  | 1 |  |
|  | Female | 52/467 (11.1) | 1.15 (0.72, 1.83) | 0.55 | 1.11 (0.69, 1.79) | 0.66 |
| **Age** | 10-11 | 6/152 (4.0) | 1 | <0.001 |  |  |
|  | 12-13 | 11/164 (6.7) | 1.75 (0.62, 4.93) |  |  |  |
|  | 14-15 | 11/179 (6.2) | 1.51 (0.54, 4.26) |  |  |  |
|  | 16-17 | 22/177 (12.4) | 3.38 (1.31, 8.74) |  |  |  |
|  | 18-19 | 38/170 (22.4) | 6.95 (2.79, 17.32) |  |  |  |
| **Education** | Below grade 7 | 20/301 (6.6) | 1 | 0.009 | 1 |  |
|  | Grade 7 | 29/286 (10.1) | 1.53 (0.83, 2.82) |  | 0.87 (0.43, 1.78) | 0.93 |
|  | Secondary or higher | 39/248 (15.7) | 2.51 (1.38, 4.56) |  | 0.93 (0.43, 2.00) |  |
| **HIV status disclosure** | Does not know status | 14/158 (8.9) | 1 | 0.25 | 1 | 0.63 |
|  | Knows status, has not disclosed | 37/373 (9.9) | 1.12 (0.56, 2.24) |  | 0.71 (0.34, 1.48) |  |
|  | Knows status, has disclosed | 32/257 (12.5) | 1.66 (0.81, 3.41) |  | 0.83 (0.38, 1.80) |  |
| **Viral load** | <1000 copies | 52/541 (9.6) | 1 |  | 1 |  |
|  | ≥1000 copies | 32/292 (11.0) | 1.11 (0.68, 1.81) | 0.67 | 1.10 (0.67, 1.83) | 0.69 |
| **SSQ score** | No red flag | 56/547 (10.2) | 1 |  | 1 |  |
|  | Red flag | 32/295 (10.9) | 1.05 (0.65, 1.70) | 0.85 | 0.98 (0.60, 1.61) | 0.94 |
| **PHQ-9 score** | Minimal | 20/163 (12.3) | 1 | 0.29 | 1 | 0.18 |
|  | Mild | 45/382 (11.8) | 0.89 (0.49, 1.62) |  | 0.84 (0.46, 1.55) |  |
|  | Moderate | 19/222 (8.6) | 0.62 (0.30, 1.26) |  | 0.60 (0.29, 1.24) |  |
|  | Moderately severe/ Severe | 4/74 (5.4) | 0.40 (0.12, 1.27) |  | 0.31 (0.09, 1.01) |  |
