# Supplementary material for: Peer-led counselling with problem discussion therapy for adolescents living with HIV in Zimbabwe: A cluster-randomised trial
Source: PLoS Med. 2022 Jan 5;19(1):e1003887. doi: 10.1371/journal.pmed.1003887 (PMC8730396; doi:10.1371/journal.pmed.1003887)
Supplement: S2 Table — (DOCX) [file pmed.1003887.s004.docx]

### S2 Table: Unadjusted intervention effect on primary and secondary outcomes at 48 weeks

|  | Zvandiri-PST | Zvandiri |  |  |
| --- | --- | --- | --- | --- |
| Binary | **n/N (%)** | **n/N (%)** | **OR (95% CI)** | **p-value** |
| *Primary outcome* | | | | |
| Viral load ≥1000 | 55/375 (14.7) | 45/379 (11.9) | 1.28 (0.81, 2.02) | 0.30 |
| *Secondary outcomes* | | | | |
| SSQ≥ 8 | 9/377 (2.4) | 40/388 (10.3) | 0.20 (0.08, 0.49) | <0.001 |
| PHQ-9 ≥10 | 11/377 (2.9) | 34/388 (8.8) | 0.31 (0.12, 0.78) | 0.01 |
| EQ-5D index score <1 | 104/377 (27.6) | 151/388 (38.9) | 0.56 (0.30, 1.05) | 0.07 |
|  |  |  |  |  |
| Continuous | **Mean (SD)** | **Mean (SD)** | **MD (95% CI)** | **p-value** |
| SSQ score | 2.22 (2.15) | 3.38 (3.02) | -1.14 (-1.82, -0.47) | 0.001 |
| PHQ-9 score | 2.40 (3.01) | 3.48 (3.83) | -1.11 (-2.04, -0.18) | 0.02 |

OR = odds ratio

MD = mean difference

All analysis adjusting for clinic as a random effect
