# Supplementary material for: Peer-led counselling with problem discussion therapy for adolescents living with HIV in Zimbabwe: A cluster-randomised trial
Source: PLoS Med. 2022 Jan 5;19(1):e1003887. doi: 10.1371/journal.pmed.1003887 (PMC8730396; doi:10.1371/journal.pmed.1003887)
Supplement: S3 Table — (DOCX) [file pmed.1003887.s005.docx]

### S3 Table: Effect modification of baseline age on trial outcomes at 48 weeks

|  | 10-14 years | | | | 15-19 years | | | |  |
| --- | --- | --- | --- | --- | --- | --- | --- | --- | --- |
|  | **Zvandiri-PST** | **Zvandiri** |  |  | **Zvandiri-PST** | **Zvandiri** |  |  |  |
| Binary | **n/N (%)** | **n/N (%)** | **AOR (95% CI)** | **p-value** | **n/N (%)** | **n/N (%)** | **AOR (95% CI)** | **p-value** | **P _interaction_** |
| *Primary outcome* | | | | | | | | | |
| Viral load ≥ 1000 | 19/180 (10.6) | 28/206 (13.6) | 0.68 (0.35, 1.31) | 0.24 | 36/195 (18.5) | 17/173 (9.8) | 2.65 (0.97, 7.23) | 0.06 | 0.01 |
| *Secondary outcomes* | | | | | | | | | |
| SSQ ≥ 8 | 0/179 (0.0) | 15/210 (7.1) | - | - | 9/198 (4.6) | 25/178 (14.0) | 0.28 (0.11, 0.69) | 0.006 | - |
| PHQ-9 ≥10 | 2/179 (1.1) | 15/210 (7.1) | 0.16 (0.03, 0.89) | 0.04 | 9/198 (4.6) | 19/178 (10.7) | 0.42 (0.16, 1.07) | 0.07 | 0.26 |
| EQ-5D index score <1 | 43/179 (24.0) | 76/210 (36.2) | 0.57 (0.27, 1.20) | 0.14 | 61/198 (30.8) | 75/178 (42.1) | 0.56 (0.26, 1.20) | 0.14 | 0.75 |
| Continuous | **mean (SD)** | **mean (SD)** | **AMD (95% CI)** | **p-value** | **mean (SD)** | **mean (SD)** | **AMD (95% CI)** | **p-value** | **P _interaction_** |
| SSQ score | 1.9 (1.86) | 2.91 (2.88) | -0.91 (-1.72, -0.10) | 0.027 | 2.47 (2.35) | 3.92 (3.10) | -1.41 (-2.27, -0.56) | 0.001 | 0.28 |
| PHQ-9 score | 1.83 (2.51) | 3.03 (3.83) | -1.20 (-2.20, -0.20) | 0.018 | 2.90 (3.33) | 4.02 (3.76) | -0.99 (-2.02, 0.03) | 0.058 | 0.44 |

AOR = odds ratio. AMD = adjusted mean difference. SD=standard deviation

All analysis adjusting for baseline value of the outcome, baseline age, and clinic as a random effect
