# Supplementary material for: Peer-led counselling with problem discussion therapy for adolescents living with HIV in Zimbabwe: A cluster-randomised trial
Source: PLoS Med. 2022 Jan 5;19(1):e1003887. doi: 10.1371/journal.pmed.1003887 (PMC8730396; doi:10.1371/journal.pmed.1003887)
Supplement: Attachment — Submitted filename: Zvandiri-PST review response.docx [file pmed.1003887.s006.docx]

**Requests from the editors:**

1. Please quote aggregate demographic details for study participants in the abstract.

*We have added gender breakdown and proportion of participants who were orphaned*

2. Please quote study dates in the abstract.

*We have added the enrolment period, January-March 2019*

3. Please add a new final sentence to the "Methods and findings" subsection of your abstract, beginning "Study limitations include ..." or similar and quoting 2-3 of the study's main limitations.

*We have added* ‘*Limitations include that cost data were not available, and that the mental health tools were validated in adult populations, not adolescents.’*

4. At line 52 and elsewhere in the elements of discussion in the paper, please quote the null findings on the study's primary endpoint prior to mentioning other findings.

*We have added this at the beginning of the discussion*

5. After the abstract, please add a new and accessible "Author summary" section in non-identical prose. You may find it helpful to consult one or two recent research papers in PLOS Medicine to get a sense of the preferred style.

*We have added an author summary*

6. Please avoid claims such as "the first", e.g., at line 339, and where needed add "to our knowledge" or similar.

*Done*

7. Please remove the information on funding from the end of the main text. In the event of publication, this will appear in the article metadata via entries in the submission form.

*Done*

8. Throughout the text, please adapt reference call-outs to the following style (noting the absence of spaces within the square brackets): "... symptoms of CMD [10,11].".

*Done throughout. This change has not been tracked.*

9. Noting reference 7, please ensure that all citations contain full access information.

*Done. We also noticed that reference 24 was identical with reference 9 so we have removed it and renumbered.*

10. Please use the journal name abbreviation "PLoS ONE" in the reference list.

*Done*

11. Please rename figure 1 "Participant flowchart" or similar.

*Done*

12. Please include a completed checklist for the most appropriate reporting guideline, e.g., CONSORT, with your revision as an attachment, labelled "S1_CONSORT_Checklist" or similar and referred to as such in the Methods section. In the checklist, please refer to individual items by section (e.g., "Methods") and paragraph number, not by line or page numbers as these generally change in the event of publication.

*A completed checklist is attached and we have added a reference to it in the methods section*

**Reviewer #1: Statistical review**

This paper reports a cluster randomised trial comparing a peer-led counselling intervention on HIV-positive adolescents with mental health problems. Generally the results are reported well. I have some minor comments on the statistical methods and reporting, which are listed below.

1. Abstract conclusions - I would recommend flipping the sentence around to emphasise the primary outcome was not met but that there were some significant secondary outcomes. The same goes for the first paragraph in the discussion.

*We have made this change to emphasise the primary outcome first.*

2. Methods page 5 - can more be said about the randomisation procedure, was it a block randomisation or was there any stratification?

*The paper says that the randomisation was 1:1 within each district, i.e. stratified by district.*

3. Methods - I'd recommend having the outcomes listed first and then the sample size section.

*We have moved the sample size section after the description of outcomes.*

4. Page 6 - the primary outcome here is given as 1000 copies per microlitre, whereas the protocol says 100 copies per millilitre - these are different by a couple of orders of magnitude - can the authors clarify? The secondary outcomes also have slightly different thresholds to those given in the protocol.

*I can’t find the first error. As far as I can see, both the paper and the protocol give the primary outcomes as ‘≥1000 copies/mL’, that is 1000 copies per millilitre. That is certainly what we meant to say.*

*For the secondary outcomes, the cutpoint values we used in our analysis are the standard ones and have been validated. The cutpoints in the protocol were non-standard. In our pre-written analytical plan we specified that we would use the validated cutpoints rather than the ones in the protocol.*

5. Results, page 9 - for the subgroup analysis I would mention the significant interaction p-value as otherwise it might be puzzling to the reader how the two non-significant subgroup p-values are evidence of a significance.

*The interaction p-value was already in the text. We have labelled it to make it clearer.*

**Reviewer #2:**

This is an excellent paper, and an important study. It compares a well-evidenced NGO program to support adolescents living with HIV, to an enhanced version with additional mental health components based on another well-evidence-based program. It is encouraging to see a commitment to building the evidence-base and optimising interventions, and to see two research groups working together (which doesn't happen as often as it should).

The original Zvandiri program has strong evidence of improving viral load suppression. The original Friendship Bench program has strong evidence of reducing common mental health disorders. This study shows that a combined program can do both, even with some complexities and challenges. This is of enormous value to programming for this group of exceptionally vulnerable adolescents.

The methods are robust, and the program delivery was done through existing government clinics, suggesting scaleability. Good measures are used. I had no comments on the methods or write-up, which are both excellent. I'm not familiar with a quadrature check for model fit, but the other analytical approaches all look strong.

The qualitative findings were extremely valuable - and it would be helpful to know whether these have contributed to any adjustments to the mental health component of the program. In retrospect, it may have been useful to have done the qualitative evaluation before the trial, to allow adjustments to be made, but it is also absolutely commendable to have accompanied the quantitative trial with qualitative study, and in the wider implementation science approach these are all iteratively feeding into program improvements.

I was struck by the importance of including caregivers, and at some point it could be of value to think about synergies between these approaches and the growing evidence-base on parenting programs for families of adolescents - which have not always shown good impacts on adolescent mental health, but have helped with some of the structural challenges mentioned, such as family budgeting, violence prevention, reducing conflict and improving family relationships.

*Thank you very much for this encouraging and helpful feedback. A major lesson learned from this study has been the importance of qualitative evaluation early in the process.*

My only minor comments are:

1. Exclusion criteria included intoxication or psychosis. There is a lot of logic to this, but in most of the countries most affected by HIV, there would be very limited other services for these adolescents, and so it would be valuable also to see whether the combined program would help even in quite extreme cases.

*We felt it was unreasonable to put young peer supporters into situations for which they were not trained. We take your point that there may be few other services available.*

2. It would be helpful to have a bit more information about the delivery of the combined program.

*This will be covered in a separate paper*

3. I know that there is a lot described in the protocol paper, but perhaps some more information could be uploaded into the supplementary material to this paper, so that it is easy for the reader to access.

*Please could you explain what information you’re referring to?*

But these are very minor. This is clearly a paper of real importance to the field, and a novel and extremely important study. Congratulations to the team.

**Reviewer #3:**

The authors conducted a clinic-randomized trial (n=60 clinics) to assess the impact of adding "training in Problem-Solving Therapy (PST)" to an established peer counselling intervention on mental health and viral suppression outcomes among adolescents with HIV.

Mental health is a predictor of adherence and retention in HIV care as well as an important health outcome in its own right. Adolescents with HIV face unique mental health challenges and this population is growing as vertically-infected children age into adolescence (e.g. Maskew et al. 2019, Lancet HIV). Whereas the (standard of care) established counselling intervention was previously shown to be effective in increasing viral suppression, it had no impact on mental health. Hence the effort to amend the intervention with PST. PST appears to be the missing link. Although the authors find no impact of the PST-augmentation on viral suppression (perhaps due to ceiling effects), they find large reductions in depression and other adverse mental health outcomes. (One can infer that relative to "no counselling", the combination of PST with the original intervention would therefore be expected to lead to improvements on both mental health and viral suppression.)

The study is important and appears carefully executed (with carefully described procedures). The combination of qualitative data is a major asset in interpreting the study findings, although I think it can be better used (see below).

General comments:

1. RATIONALE. I think the study would be strengthened by a clearer explication of the intervention rationale (why PST?). The content of PST-training (Friendship Bench) is described. However, what are the theoretical constructs that PST is designed to address? And how did the authors identify these constructs (based on their prior work) as needing to be addressed? My initial sense is that the intervention was designed to (a) give people a sense of agency and control in their lives and (b) to provide cognitive tools to assist with problem solving and give opportunities for practice using these tools. I infer that a major source of mental morbidity is a sense of lack of control which may be exacerbated by having HIV. And building up a sense of agency as well as problem solving skills could theoretically support ART adherence too. (But these were my own speculations based on the paper. Further details here would help the reader understand the nature of the problem that needs to be solved. In fact, the bottom of p10 suggests a different interpretation based on coping and validation rather than agency.)

*Thank you for this thoughtful feedback. The points you have made are interlinked but I have tried to address them in order as they come up.*

*PST is based in cognitive behavioural therapy and is designed to improve problem solving skills, develop resilience and adaptive skills, and enhance agency. By this means, Friendship Bench has improved mental health and other health outcomes in adults. We expected it to have the same effect in adolescents. We have added details on the theoretical framework in the introduction.*

2. FIDELITY, FEASIBILITY, and ACCEPTABILITY. There is a bit of whiplash as the authors present large intervention effects and then present qualitative data indicating that the intervention was actually rather difficult to implement (low feasibility, acceptability) and had low fidelity in implementation. So, which is it? If the data indicate that the intervention was not really implemented faithfully, should we interpret this as evidence that the highly significant effects on mental health occurred by chance? Or should we rather interpret this as evidence that the particular components of the intervention that were implemented turned out to be important in this context?

I love the combination of qual and quant. But as it is, my feeling is that the qual results undercut the quant findings, rather than adding validity and nuance. The qual results are framed as providing information on "what was wrong about the intervention implementation." But this is immaterial. The question in the reader's mind after the quantitative results is "what was RIGHT about the intervention implementation", i.e. what led to the big mental health impacts observed?

I think the discussion of fidelity would be greatly improved if it were organized around key questions that guide interpretation of the quantitative results. E.g., (a) can the authors provide some evidence that study participants in the treatment group actually received something different than participants in the control group? (b) can the authors be more explicit about WHAT exactly was different? E.g. if it seems that the earlier stages of PST were emphasized, then that's valuable to know. (c) How do the findings on fidelity shape interpretation of the theoretical model? i.e. if the intervention was designed to affirm the experiences of subjects, to build locus of control, and to build cognitive problem-solving skills… but if only the first of these was actually implemented, then that is pretty important for interpretation. But the authors should set this up ex ante that the intervention was designed to target different constructs. And (d) how can this evidence guide future development of the intervention and inform scale-up?

*The reason for the ‘whiplash effect’ is that the intervention as originally conceived turned out to be unfeasible in practice. The intervention was not implemented faithfully, as such. It was adapted by the trained CATS into something else, which we have called ‘problem discussion therapy’ in the paper, distinct from the problem solving therapy that was the original plan. The effects on mental health did not occur by chance, they were a result of the problem discussion therapy. We felt it was important to emphasise this, because our results should not be taken as evidence that PST is effective for adolescents. That is why the qualitative results appear to undercut the quantitative.*

Smaller stuff:

ABSTRACT

3. I found the intervention content and rationale to be unclear. The abstract states that the comparison was between peer-counselling and peer-counselling where the counsellor was trained in Problem-Solving Therapy. However, it would be helpful to know: (a) how did PST differ from the standard of care counselling; (b) did the counsellors actually deliver PST or were they just trained in PST; (c) what was the theoretical motivation for PST relative to standard of care, i.e. what was the problem that PST was introduced to solve?

*This is similar to the general comments above. PST was designed to improve resilience, problem solving skills and autonomy. The counsellors did not actually deliver PST because they found it was unfeasible and unhelpful. The CATS used their understanding of the aim of the intervention and adapted it into something more appropriate.*

4. In the description of results, it is unclear what 14.7% and 11.9% refer to. Are these prevalences of viral non-suppression? Or percent reductions in non-suppression? Or percentage point reductions in non-suppression?

*Prevalence of viral non-suppression. We have added this in the text*

5. Conclusion "Active involvement of caregivers and strengthened referral structures could increase feasibility and effectiveness" doesn't seem to follow from the study results.

*These were conclusions from the qualitative findings, for example themes 1 and 3 in Table 5. We presented evidence from CATS on their experience of involving caregivers. The CATS also reported that they required a robust referral structure for times when they were unable to cope with participants’ needs.*

METHODS

6. "An a-priori decision was made to adjust for baseline values of the relevant outcome measure and for key variables that were deemed imbalanced between arms at baseline or were associated with missing outcome data." The authors should also present crude/unadjusted results. And the decision rules for how variables were determined to be "key" and therefore adjusted for should be stated.

*These would be additional results that were not prespecified in the analytical plan.*

*There were no specific decision rules for how to decide which variables to adjust for. It depends on the extent of the imbalance, the relevance of that variable to the outcomes, ability to accurately measure the characteristic, and so on. The important thing is that the decision was made prior to analysis and therefore blind to study findings. The only variable that was adjusted for, apart from baseline value of the outcome, was age.*

RESULTS

7. "The proportion of participants with an EQ-5D score below 1 was slightly lower in the Zvandiri-PST arm than in the Zvandiri arm (27.6% vs 38.9%; AOR=0.56; 95%CI 0.31, 1.03; p=0.06)." The term "slightly lower" is inaccurate. The PST arm proportion was >40% lower than control! The p-value was "slightly higher" than conventional cut-offs for statistical significance (but the CI is all that matters anyway).

*Thank you for pointing this out. We have corrected it.*

CONCLUSIONS

8. "Our finding that PST, as originally envisaged, was not appropriate or feasible for an adolescent population is in line with the literature." I don't understand. It feels like the authors are saying that the intervention didn't work. But it DID work! (unless I misunderstand)

*The intervention as originally envisaged was not appropriate or feasible. It was modified in practice. The modified version worked.*

Final word:

9. The availability of both qualitative and quantitative (trial) data is a major asset to this study. However, while each component (quant and qual) was well done, the current manuscript does not integrate the two very well. It feels like a trial results paper on the success of an intervention, followed by a qual study on how the intervention failed. This contradiction led to a great deal of confusion in this reader as to whether the trial results were credible and missed opportunities to explicate the reasons for the large trial effects. A cluster-randomized trial is higher quality evidence (per GRADE standards) and should dictate the overall conclusions of the study. I strongly recommend the authors cut the qual section by 50% and use the qual section to answer very specific questions that will help the reader interpret what went well (leading to large effects) in the intervention and what components of the PST intervention to emphasize going forward if it is brought to scale.

*I can understand why the paper gives that impression. We have tried to address these specific questions. The qualitative analysis focuses on three themes: (1) how the fact adolescents’ problems are relational and poverty-related impeded fidelity to the intervention model since problem-solving was not possible; (2) the aspects of the intervention that were beneficial, i.e. peer-led problem discussion and empathic listening; (3) what needs to be added to the intervention, specifically caregiver involvement and a mental health care support network.*
