# Supplementary material for: Peer-led counselling with problem discussion therapy for adolescents living with HIV in Zimbabwe: A cluster-randomised trial
Source: PLoS Med. 2022 Jan 5;19(1):e1003887. doi: 10.1371/journal.pmed.1003887 (PMC8730396; doi:10.1371/journal.pmed.1003887)
Supplement: Attachment — Submitted filename: Zvandiri-PST review response 181121.docx [file pmed.1003887.s007.docx]

*Dear editors,*

*Thank you for this second review. Please find below our response to each point.*

**Requests from the editors:**

Noting reviewer 3's comments, we ask you to explain how the modification of the intervention fits in with the conduct and findings of the trial - did this happen before or, as apparently the case, during the study, for example? We reserve judgement about the suggested restructuring of the paper pending resolution of the previous question.

*The modification of the intervention ocurred during the study. We, the investigators, discovered the extent of it following analysis of the process evaluation. It was not formative research, and for this reason we suggest keeping the trial results before the process evaluation results.*

*The dialogue about this study with Reviewer 3 has raised some important questions about, for instance, what replicability means for a therapy that is intrinsically linked to individual context, and how to describe an intervention that was adapted away from the original protocol towards something that was in fact more appropriate (and hence effective). It also highlights the importance of formative research and process evaluation. We would like to suggest that if the paper is published, Reviewer 3 could write a letter or opinion piece accompanying it to present their point of view.*

In the response to referees you mention that the intervention delivered was "problem discussion therapy" rather than "problem-solving therapy" per se, and it seems that judicious changes need to be made in the text, for example around line 140, to reflect this.

*We have amended the text to “Participants at the Zvandiri-PST clinics received … ~~PST~~ additional sessions based on the CATS’ PST training.”*

We ask you to adapt the title to better match journal style, and suggest: "Peer-led counselling with problem discussion therapy for adolescents living with HIV in Zimbabwe: A cluster-randomised trial".

*Done.*

In the abstract and elsewhere in the paper, please soften the language describing secondary outcome findings, e.g., "... evidence of an apparent effect"

*Done.*

In the author summary, please present the primary outcome findings before those of the secondary outcomes.

*Done.*

Please avoid claims of "the first", e.g., at line 92, and where necessary add "to our knowledge" or similar.

*We have added ‘To our knowledge’.*

Please substitute "sex" for "gender" where appropriate, e.g., in table 2.

*Done*

Please update reference 23.

*Done.*

**Comments from the reviewers:**

**Reviewer #1:**

Thank you to the authors for addressing my previous comments. Regarding the primary endpoint cut-point, this is denoted as 1000 copies/mL on page 2 line 35 and 1000 copies/µl (which I understand as microlitre rather than millilitre) on page 8 line 170. Otherwise I am happy with the paper and have no issues to raise.

*Thank you, this has been corrected.*

**Reviewer #2:**

I am happy with the authors responses to the reviewer and editor comments. Recommend acceptance. Lucie Cluver.

*Thank you.*

**Reviewer #3:**

Thank you for the opportunity to re-review this paper. I appreciate the clarifications that the authors have made in response to my comments. However, several of the underlying issues remain unaddressed.

1) The authors have clarified that "The intervention as originally envisaged was not appropriate or feasible. It was modified in practice. The modified version worked."

In light of authors' feedback, it is not clear what the intervention was. The intervention is described in the abstract as "training peer counsellors in PST". Yet if PST was not delivered, then it is not at all clear that training counsellors in PST was the key element of the intervention. What WAS the intervention? And how would someone replicate this intervention in another setting? Inasmuch as research is about creating generalizable knowledge, the intervention needs to be well-defined in order for the reader to draw any conclusions about it. This is a major limitation of the study.

*The difference between the two trial arms was that in one arm peer counsellors had PST training and in the other arm they did not, so by definition that was the intervention. Alternatively you could consider that the intervention is what the participant actually received. In that sense, every participant received a unique, personalised talking therapy from a lay counsellor.*

*I agree that that an intervention needs to be well-defined in order for the reader to draw conclusions about it, and that is why the paper includes a qualitative section describing the intervention implementation in detail. We showed that the training (the intervention), in context, led to an adaptation of therapy which in turn led to improved mental health outcomes. I think this paper raises important issues about generalisability and replicability by demonstrating how a training intervention of talk therapy, such as this, is deeply affected by its context.*

I still believe the study is interesting and important. But...
(A) The authors should change how they present the intervention. They might consider labelling the study a "pilot" of a new, situationally-adapted mental health counseling intervention based on PST principles and training. They may also specify that this was a community-engaged intervention development approach, with peer counselors providing ongoing feedback and shaping the design of the intervention as it was implemented. And that the goals of the intervention were to engage counsellors in an effort to design wraparound mental health services, in which they had discretion to develop their own protocols.

*This would be a post hoc attempt to suggest that we had planned everything all along. In reality we did not set out to engage counsellors in a co-creation process (although it would have been a good idea). Our intention was to trial PST. The process evaluation revealed that what actually happened was more complex. I think this is very valuable. Among other things, our study highlights the critical importance of a thorough process evaluation to accompany any trial, particularly a trial of a talking therapy. Without the process evaluation, we and everyone else would have concluded that PST is effective at improving mental health among ALHIV.*

(B) The authors should invert the paper, describing the intervention development process, process outcomes, and intervention content (as implemented) first, BEFORE showing the data on impact of the intervention on the primary and secondary outcomes. As currently organized, it was hard for the reader to interpret the results without a clear understanding of the intervention first.

*It is typical to present trial results before the process evaluation, although the results of a process evaluation always have implications for interpretation of trial findings. We will leave it to the editors to make the decision on whether the order of the two sections should be swapped. Chronologically, the trial results came first.*

(C) Relatedly, the role of the qualitative data collection and analysis in the overall project needs to be specified. (I.e. the mixed methods design) Inasmuch as the qualitative research guided intervention development, it should be labeled as "formative research" and presented before the data on the trial results. Alternatively, if it was "explanatory research" of process outcomes, then it should be labeled as such and STILL presented before the other outcomes.

*The qualitative research was explanatory research of process outcomes. The first sentence of the qualitative methods section says “To better understand how the CATS experienced implementing the intervention, including its feasibility and any necessary modifications, we collected qualitative data to capture concurrent and retrospective accounts of the CATS”. I think this clearly explains the role of the qualitative data collection and analysis.*

2) The authors did not respond to my request for further information on the theoretical constructs that the friendship bench intervention was designed to target, why these were relevant to the adolescent HIV population, and which of these constructs the ADAPTED version of the intervention (i.e. not PST) still targeted. Given that there was poor fidelity to the planned intervention, understanding what constructs were actually targeted successfully and which were not is critical for any kind of generalizability to other settings. A lot of this comes out in the qualitative results. But it would be advantageous to spell out at the beginning of the paper / in the methods section what constructs were being targeted.

*Constructs the* *Friendship Bench program focuses on are exploration and understanding of the clients’ situational contexts through talk therapy, mentalization, positive relational experience through being listened to, and intrapersonal growth towards strength and ability through goal-oriented learning. We have added this to the introduction.*

*As explained in the paper, the CATS experienced difficulties in the first steps of the PST approach when helping their clients to choose a problem to focus on. Relational experiences and being listened to are important constructs that were successfully deployed, which is explained in the qualitative findings.*

3) The authors did not adequately address my comment #6. I requested crude/unadjusted results from the RCT. These are always appropriate whether pre-specified or not. Further, I requested information on how the authors determined what variables to include in the adjusted model. In the reply, it is unclear whether in fact there were pre-specified decision rules or not. They write "there were no specific decision rules" and then "the decision was made prior to analysis". If there was a pre-analysis protocol defining what was adjusted for, the authors can link to that. But if they chose variables as they went along based on their associations with the trt and outcome non-response, then they should show robustness checks with other adjustment variables. Basically, they need to convince the reader that they did not "choose" their preferred specification and that the benefits of randomization were preserved.

*The crude results have now been presented in Supplementary Table 2. They are very similar to the adjusted results already shown.*

*The pre-written analytical plan is attached, and we have referred to it as an appendix in the paper. The section on ‘Baseline comparability of randomised groups’ says:*

*“Baseline characteristics of enrolled participants will be compared between treatment arms and also reported overall, summarised using mean and standard deviation, median and interquartile range or numbers and proportions as appropriate. No significance testing will be done as any differences are due to chance if randomisation was correctly applied. …*

*Among those enrolled, the following baseline characteristics will be compared by arm:*

- *age*
- *gender*
- *in school*
- *orphanhood status*
- *HIV viral load at enrolment*
- *aware of HIV status*
- *ever disclosed HIV status*
- *mean SSQ score*
- *mean PHQ-9 score*

*Any variables for which there is a substantial imbalance will be noted so that final analysis can take this into account.”*

*We assessed imbalance in these 9 variables and decided to adjust for age.*

I remain convinced that this is an important study. However, I cannot recommend it for publication in its current form based on the methodological concerns highlighted above.

*I appreciate the time you have taken to review this paper and I hope the changes we have made are sufficient.*
