# Supplementary material for: Peer-led counselling with problem discussion therapy for adolescents living with HIV in Zimbabwe: A cluster-randomised trial
Source: PLoS Med. 2022 Jan 5;19(1):e1003887. doi: 10.1371/journal.pmed.1003887 (PMC8730396; doi:10.1371/journal.pmed.1003887)
Supplement: Attachment — Submitted filename: response.docx [file pmed.1003887.s008.docx]

Reviewer #3:

I thank the authors for their thoughtful responses and clarifications. I appreciate the addition

of the crude RCT results. And I think the authors' justifications regarding manuscript

organization are reasonable.

A final suggestion: to avoid the feeling of whiplash that I experienced in reading this article

the first time, I would urge the authors to assist the reader a bit by defining the intervention a

bit more broadly than PST, e.g. "discussion based therapy by peer counsellors trained in

PST", and foreshadowing that there was no further intervention to ensure that peer

counselors were staying close to PST protocols and that the counselors were free to use

and interpret their training as they saw fit. ...or something like that. So that when you get to

the process outcomes, the focus is on "why did it work" rather than on "looks like it didn't

really work".

We have added a line about the qualitative findings to the Author Summary.
